# Genome assembly of wild loquat (*Eriobotrya japonica*) and resequencing provide new insights into the genomic evolution and fruit domestication in loquat

**DOI:** 10.1093/hr/uhac265

**Published:** 2022-12-02

**Authors:** Danlong Jing, Xinya Liu, Qiao He, Jiangbo Dang, Ruoqian Hu, Yan Xia, Di Wu, Shuming Wang, Yin Zhang, Qingqing Xia, Chi Zhang, Yuanhui Yu, Qigao Guo, Guolu Liang

**Affiliations:** Key Laboratory of Horticulture Science for Southern Mountains Regions of Ministry of Education, College of Horticulture and Landscape Architecture, Southwest University, Chongqing 400715, China; Academy of Agricultural Sciences of Southwest University, State Cultivation Base of Crop Stress Biology for Southern Mountainous Land, Chongqing 400715, China; Key Laboratory of Horticulture Science for Southern Mountains Regions of Ministry of Education, College of Horticulture and Landscape Architecture, Southwest University, Chongqing 400715, China; Academy of Agricultural Sciences of Southwest University, State Cultivation Base of Crop Stress Biology for Southern Mountainous Land, Chongqing 400715, China; Key Laboratory of Horticulture Science for Southern Mountains Regions of Ministry of Education, College of Horticulture and Landscape Architecture, Southwest University, Chongqing 400715, China; Academy of Agricultural Sciences of Southwest University, State Cultivation Base of Crop Stress Biology for Southern Mountainous Land, Chongqing 400715, China; Key Laboratory of Horticulture Science for Southern Mountains Regions of Ministry of Education, College of Horticulture and Landscape Architecture, Southwest University, Chongqing 400715, China; Academy of Agricultural Sciences of Southwest University, State Cultivation Base of Crop Stress Biology for Southern Mountainous Land, Chongqing 400715, China; Key Laboratory of Horticulture Science for Southern Mountains Regions of Ministry of Education, College of Horticulture and Landscape Architecture, Southwest University, Chongqing 400715, China; Academy of Agricultural Sciences of Southwest University, State Cultivation Base of Crop Stress Biology for Southern Mountainous Land, Chongqing 400715, China; Key Laboratory of Horticulture Science for Southern Mountains Regions of Ministry of Education, College of Horticulture and Landscape Architecture, Southwest University, Chongqing 400715, China; Academy of Agricultural Sciences of Southwest University, State Cultivation Base of Crop Stress Biology for Southern Mountainous Land, Chongqing 400715, China; Key Laboratory of Horticulture Science for Southern Mountains Regions of Ministry of Education, College of Horticulture and Landscape Architecture, Southwest University, Chongqing 400715, China; Academy of Agricultural Sciences of Southwest University, State Cultivation Base of Crop Stress Biology for Southern Mountainous Land, Chongqing 400715, China; Key Laboratory of Horticulture Science for Southern Mountains Regions of Ministry of Education, College of Horticulture and Landscape Architecture, Southwest University, Chongqing 400715, China; Academy of Agricultural Sciences of Southwest University, State Cultivation Base of Crop Stress Biology for Southern Mountainous Land, Chongqing 400715, China; Key Laboratory of Horticulture Science for Southern Mountains Regions of Ministry of Education, College of Horticulture and Landscape Architecture, Southwest University, Chongqing 400715, China; Academy of Agricultural Sciences of Southwest University, State Cultivation Base of Crop Stress Biology for Southern Mountainous Land, Chongqing 400715, China; Key Laboratory of Horticulture Science for Southern Mountains Regions of Ministry of Education, College of Horticulture and Landscape Architecture, Southwest University, Chongqing 400715, China; Academy of Agricultural Sciences of Southwest University, State Cultivation Base of Crop Stress Biology for Southern Mountainous Land, Chongqing 400715, China; Key Laboratory of Horticulture Science for Southern Mountains Regions of Ministry of Education, College of Horticulture and Landscape Architecture, Southwest University, Chongqing 400715, China; Academy of Agricultural Sciences of Southwest University, State Cultivation Base of Crop Stress Biology for Southern Mountainous Land, Chongqing 400715, China; Key Laboratory of Horticulture Science for Southern Mountains Regions of Ministry of Education, College of Horticulture and Landscape Architecture, Southwest University, Chongqing 400715, China; Academy of Agricultural Sciences of Southwest University, State Cultivation Base of Crop Stress Biology for Southern Mountainous Land, Chongqing 400715, China; Key Laboratory of Horticulture Science for Southern Mountains Regions of Ministry of Education, College of Horticulture and Landscape Architecture, Southwest University, Chongqing 400715, China; Academy of Agricultural Sciences of Southwest University, State Cultivation Base of Crop Stress Biology for Southern Mountainous Land, Chongqing 400715, China; Key Laboratory of Horticulture Science for Southern Mountains Regions of Ministry of Education, College of Horticulture and Landscape Architecture, Southwest University, Chongqing 400715, China; Academy of Agricultural Sciences of Southwest University, State Cultivation Base of Crop Stress Biology for Southern Mountainous Land, Chongqing 400715, China

## Abstract

Wild loquats (*Eriobotrya japonica* Lindl.) provide remarkable genetic resources for studying domestication and breeding improved varieties. Herein, we generate the first high-quality chromosome-level genome assembly of wild loquat, with 45 791 predicted protein-coding genes. Analysis of comparative genomics indicated that loquat shares a common ancestor with apple and pear, and a recent whole-genome duplication event occurred in loquat prior to its divergence. Genome resequencing showed that the loquat germplasms can be distinctly classified into wild and cultivated groups, and the commercial cultivars have experienced allelic admixture. Compared with cultivated loquats, the wild loquat genome showed very few selected genomic regions and had higher levels of genetic diversity. However, whole-genome scans of selective sweeps were mainly related to fruit quality, size, and flesh color during the domestication process. Large-scale transcriptome and metabolome analyses were further performed to identify differentially expressed genes (DEGs) and differentially accumulated metabolites (DAMs) in wild and cultivated loquats at various fruit development stages. Unlike those in wild loquat, the key DEGs and DAMs involved in carbohydrate metabolism, plant hormone signal transduction, flavonoid biosynthesis, and carotenoid biosynthesis were significantly regulated in cultivated loquats during fruit development. These high-quality reference genome, resequencing, and large-scale transcriptome/metabolome data provide valuable resources for elucidating fruit domestication and molecular breeding in loquat.

## Introduction

Loquat (*Eriobotrya japonica* Lindl.), a subtropical evergreen fruit tree, is one of the most popular fruits in the world, and belongs to the family Rosaceae, which includes apple, pear, strawberry, and other economically important species [1, 2]. Loquat, which originated in China, has been cultivated for more than 2000 years and is now broadly cultivated in over 30 countries worldwide, such as China, Japan, India, Spain, Italy, Turkey, South Africa, USA, Brazil, and Australia [2, 3]. Its fruits are rich in nutrients, including carbohydrates, amino acids, vitamins, organic acids, and minerals, in addition to which they taste delicious [4–6]. Furthermore, loquat fruits are used in food industries to prepare juices, wines, syrups, and jams [2, 7]. However, the evolutionary and domestication history of loquat has long been controversial. At present, wild loquats provide a multitude of valuable genetic resources for studying systematic evolution and breeding improved varieties, because they are mainly subjected to natural selection and have only a slight effect of artificial selection.

Analysis of genetic variations between wild and cultivated germplasms not only contributes to a better understanding of the domestication of crop species but also provides an effective way to identify functional genes, which could be better utilized in genetic improvement of breeding practices [8–13]. In particular, we previously found that wild loquats often adapt to marginal environments and acquire beneficial traits, such as larger panicles, larger fruit clusters, and superior stress tolerance [14]. However, due to a complex genetic background and long cultivation history, the whole-genome genetic variations between wild and cultivated loquats remain largely unclear. Therefore, there is a need for the genome assembly and whole-genome resequencing of wild loquat, which would help identify effective ways to uncover the evolutionary and domestication processes affecting the genome of loquat plants, as well as facilitate studies in loquat biology.

Here we reveal the first chromosome-level genome of wild loquat and investigate its genome evolution. Whole-genome resequencing of wild and cultivated germplasms was performed to reveal their genetic diversity and population structure and further identify selective sweep signatures in the genome during loquat domestication. Accordingly, we also identified the key regulatory genes and metabolic pathways that are associated with fruit quality and size. These resources would facilitate genetic improvement of loquat plants and enhance the understanding of genomic diversity, the domestication process, and the formation of fruit flavor in loquat.

## Results

### Assembly of wild loquat GZ-23 genome and annotation

**Figure 1:**
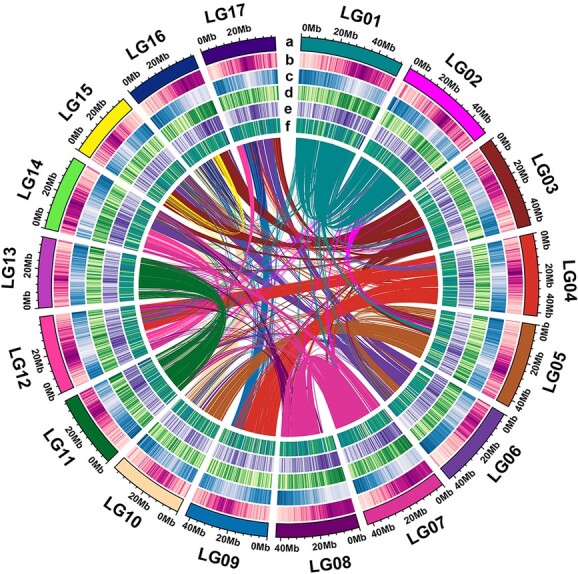
Features of the wild loquat genome assembly. (a) Circular representation of the 17 pseudomolecules. (b) Transposable element density. (c) Gene density. (d) GC content. (e) LTR retrotransposon. (f) Non-coding RNA density. The inner lines show the syntenic blocks in the wild loquat genome.

The genome of a wild loquat tree, which contains 17 distinct pairs of chromosomes (2*n* = 34), was investigated (Supplementary Data Fig. S1). We obtained an estimated genome size of 737.06 Mb using *k*-mer analysis, which was consistent with the size of ~760 Mb using flow cytometry (Supplementary Data Table S1 and Supplementary Data Fig. S1). Subsequently, a total of 159.8 Gb of raw data were generated using Nanopore sequencing (Supplementary Data Table S2). Then, 152.6 Gb of clean long reads were obtained, which represented ~198.5× coverage depth of the genome, with an N50 read length of 31.6 kb (Supplementary Data Table S3). Following this, an initial wild loquat genome assembly was constructed using the Nanopore reads. We further used Illumina paired-end and Hi-C interaction maps to perform error correction of the initial assembly and generate a final assembly of 783.7 Mb anchoring to 17 chromosome-scale scaffolds (hereafter called chromosomes LG1–LG17) (Fig. 1). The scaffold N50 and contig N50 sizes of the wild loquat GZ-23 were 41.8 and 3.9 Mb, respectively (Table 1). Then, ~8 Gb of RNA sequencing (RNA-seq) data were used to evaluate the quality of the assembly from six different loquat tissues (leaf, stem, flower, fruit, seed, and root); 98.7% of the RNA-seq transcripts were mapped back to the assembly (Supplementary Data Table S4). Following this, 449 (98.03%) of the 458 ultraconserved core eukaryotic genes were identified in the wild loquat genome assembly using the Core Eukaryotic Genes Mapping Approach (CEGMA [15]). Meanwhile, Benchmarking Universal Single Copy Orthologs (BUSCO), with a database of 1614 conserved core genes, was used to evaluate the assembly quality, and it showed that 1586 (98.27%) core genes were found to be complete in our assembly (Table 1), of which 65.12% were single copies and 33.15% were duplicated copies. Taken together, these results suggest that the genome assembly of wild loquat is of high quality.

The methods of *ab initio* gene prediction, homology comparison, and transcriptome-based prediction were combined to annotate protein-coding genes. We predicted a total of 45 791 genes in the wild loquat genome assembly. Among these predicted genes, the average gene length and coding sequence length were 3315.7 and 1246.6 bp, respectively (Table 1). Importantly, the gene densities were non-homogeneously distributed, with an increase toward the ends of the chromosomes (Fig. 1). In total, 468.8 Mb (59.75% of the genome assembly) of repetitive sequences were identified (Supplementary Data Table S5). Among these repetitive sequences, retroelements (Class I elements) were predominant and covered 399.5 Mb (50.92%), of which 386.4 Mb (49.24%) were long terminal repeat (LTR) retrotransposons. Meanwhile, DNA transposons (Class II elements) covered 69.3 Mb (8.83%) of the assembly. Overall, transposable element densities were higher toward the pericentromeric regions of the chromosomes (Fig. 1). Furthermore, 5381 rRNAs, 765 tRNAs, and 129 miRNAs were identified (Supplementary Data Table S6).

**Table 1:** Summary statistics of wild loquat genome assembly and annotation.

Assembly feature	Value
Assembly size	783.7 Mb
Number of scaffolds	230
Length of largest scaffolds	52.8 Mb
Scaffold N50 size	41.8 Mb
Number of contigs	526
Length of largest contig	16.9 Mb
Contig N50 size	3.9 Mb
GC content	37.76%
Sequences anchored to chromosomes	99.88%
CEGMA complete percentage in assembly	98.03%
BUSCO complete percentage in assembly	98.27%
Gene number	45 791
Average gene length	3315.7 bp
Average coding sequence length	1246.6 bp

### General characteristics of genome evolution

**Figure 2:**
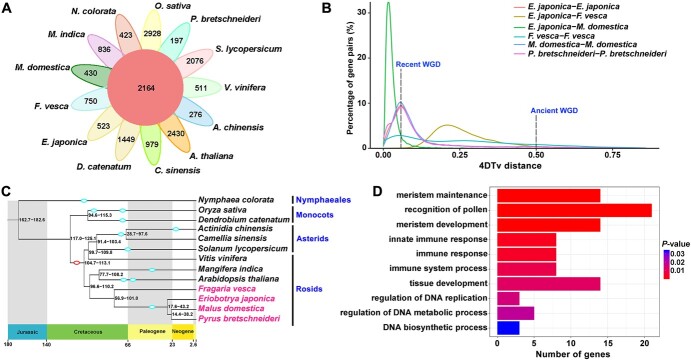
Gene family, WGD, and gene expansion in a wild loquat genome. (A) Gene family clusters in wild loquat and 12 other representative angiosperms. (B) Distribution of 4-fold degenerate site (4DTv) distances of duplicate gene pairs in loquat, strawberry, apple, and pear. (C) Phylogenetic tree and divergence times of wild loquat and 12 other angiosperms. The WGD events are indicated by circles. The paleo-hexaploidization event is indicated by a red circle. (D) Biological process categories of the expanded gene families in wild loquat.

To obtain the characteristics and evolution of the loquat genome, a comparative genomic analysis was performed using loquat and 12 representative angiosperms, including one basal angiosperm, *Nymphaea colorata*; two monocots, *Oryza sativa* and *Dendrobium catenatum*; three asterids, *Solanum lycopersicum*, *Camellia sinensis*, and *Actinidia chinensis*; and six rosids, *Malus domestica*, *Pyrus bretschneideri*, *Fragaria vesca*, *Arabidopsis thaliana*, *Mangifera indica*, and *Vitis vinifera*. Compared with those from the 12 other angiosperms, orthologous clustering of the predicted loquat proteins showed that 92% (42 150) of the protein-coding genes were assigned to 20 668 gene families, with an average of 2.04 genes per family; 523 gene families were unique to loquat, more than that in *M. domestica* (430), *P. bretschneideri* (197), and *V. vinifera* (511), but fewer than that in *F. vesca* (750) (Fig. 2A and Supplementary Data Table S7). Specific gene families in loquat were mainly involved in the biological processes of regulation of biological quality, hormone levels, localization, transport, and auxin polar transport, in addition to meristem structural organization, anatomical structure arrangement, homologous chromosome segregation, sister chromatid segregation, and the attachment of spindle microtubules to the kinetochore (Supplementary Data Fig. S2).

The distributions of 4-fold degenerate sites (4DTv) and synonymous substitutions (*K*_s_) of paralogous genes were used to detect whole-genome duplication (WGD) events (Supplementary Data Figs S3 and S4A). The results showed that the loquat genome has two significant peaks in both 4DTv and *K*_s_ distributions (*E. japonica*_*E. japonica*), suggesting two WGD events have occurred in the loquat genome. Furthermore, we found that the first peak (at ~0.15) of *K*_s_ distribution indicated the gamma polyploidy event in core eudicots, while the second peak (at ~0.05) revealed that loquat underwent a recent WGD event (Supplementary Data Fig. S4A); the *K*_s_ distribution of syntenic gene pairs was similar to that seen in pear (Supplementary Data Fig. S4B). The 4DTv distribution of all duplicate gene pairs indicated that the recent WGD events are shared by loquat, apple, and pear (Fig. 2B). As reported in apple and pear, the recent WGD in loquat must have occurred 30–45 million years ago (MYA) [16, 17], while the ancient WGD must have resulted from an acknowledged paleo-hexaploidization event, which took place ~120–130 MYA [18, 19].

Based on 1028 single-copy orthologs from the genomes of the 13 species, we constructed a phylogenetic tree to estimate species divergence time. Our results showed that loquat shared a common ancestor with apple and pear, ~17.6–43.2 MYA, but the divergence of these three species occurred after a recent WGD event (Fig. 2C). Furthermore, comparative and evolutionary analyses of gene families in the 13 plant species were performed. In loquat, 104 gene families, comprising 1765 genes, exhibited significant expansion compared with that of the last common ancestor, but only one gene family, comprising 24 genes, showed contraction (Supplementary Data Fig. S5). Consistently, Gene Ontology (GO) enrichment analysis indicated that the expanded gene families in loquat were mainly classified into the biological processes of pollen recognition, tissue development, meristem development, meristem maintenance, immune system process, immune response, innate immune response, and regulation of DNA metabolic process (Fig. 2D). Meanwhile, Kyoto Encyclopedia of Genes and Genomes (KEGG) pathway enrichment analysis showed that the expanded loquat gene families were mainly involved in galactose metabolism, cyanoamino acid metabolism, ascorbate and aldarate metabolism, glyoxylate and dicarboxylate metabolism, amino sugar and nucleotide sugar metabolism, nitrogen metabolism, biosynthesis of unsaturated fatty acids, phenylalanine metabolism, betalain biosynthesis, and glucosinolate biosynthesis (Supplementary Data Fig. S6). However, the biological process of the contracted gene family was only involved in protein phosphorylation (Supplementary Data Fig. S7).

### Loquat germplasm diversity

We performed whole-genome resequencing of 26 typical cultivated loquats, representing the cultivated germplasms in different areas of the globe, and 11 wild germplasms collected from southwest China. Across the 37 accessions, a total of 690.11 Gb of clean reads were obtained, with an average depth of 22.49× and average coverage of 98.65% of the wild loquat genome (Supplementary Data Table S8). Ultimately, 10 978 138 high-quality single-nucleotide polymorphisms (SNPs), with an average of 14.01 SNPs per kilobase, were identified. Meanwhile, 919 496 insertions, and 944 676 deletions were identified. Among these variants, 17 197 insertions and 21 890 deletions were located in the genic regions (Supplementary Data Table S9).

**Figure 3:**
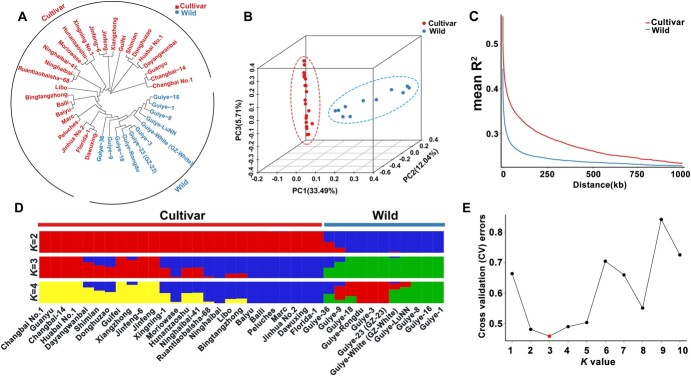
Genomic diversity of wild and cultivated loquats. Twenty-six typical cultivated germplasms and 11 wild germplasms were sampled for the analyses. (A) Phylogenetic tree of 37 loquat accessions based on SNPs. (B) PCA analysis of wild and cultivated loquats using SNP markers. (C) LD in wild and cultivated groups. (D) STRUCTURE analysis of loquat accessions for *K* = 2, 3, and 4. Each color represents one population, and each color segment length in each vertical bar indicates the proportion is contributed by ancestral populations. (E) *K*-value estimation.

These high-quality SNPs were used to carry out phylogenetic analysis and principal component analysis (PCA) of wild and cultivated loquats. The phylogenetic and PCA analyses showed that these germplasms were clustered into two distinct groups, with cultivated germplasms classified into one group and wild germplasms into the other (Fig 3A and B). The nucleotide diversity (π) of wild loquats here was 2.28 × 10^−3^, which was higher than that of the cultivated loquats (1.44 × 10^−3^). Linkage disequilibrium (LD) analyses within wild and cultivated loquats showed that the LD in wild loquats decayed to basal levels at ~750 kb, while the LD in cultivated loquats decayed to basal levels at ~1000 kb (Fig. 3C). The LD decay of the wild loquats was faster than that of cultivated loquats. The mean values of expected heterozygosity and observed heterozygosity in cultivated loquats were significantly lower than those in wild loquats (Supplementary Data Table S10). We then further performed population structure analysis using the software STRUCTURE. When *K* = 2 (the number of predefined genetic clades), all individuals were clearly subdivided into the two specific clades of wild and cultivated germplasms. Three individuals of wild germplasms (Guiye-36, Guiye-9, and GZ-White) showed a mixture of genetic components of cultivated germplasms (Fig. 3D); this might be related to gene flow between wild and cultivated germplasms. Meanwhile, three genetic clades (*K* = 3) represent the best model (Fig. 3E). When *K* = 3, the cultivated germplasms were divided into two distinct clades and 11 commercial cultivars experienced allelic admixture (Fig. 3D).

**Figure 4:**
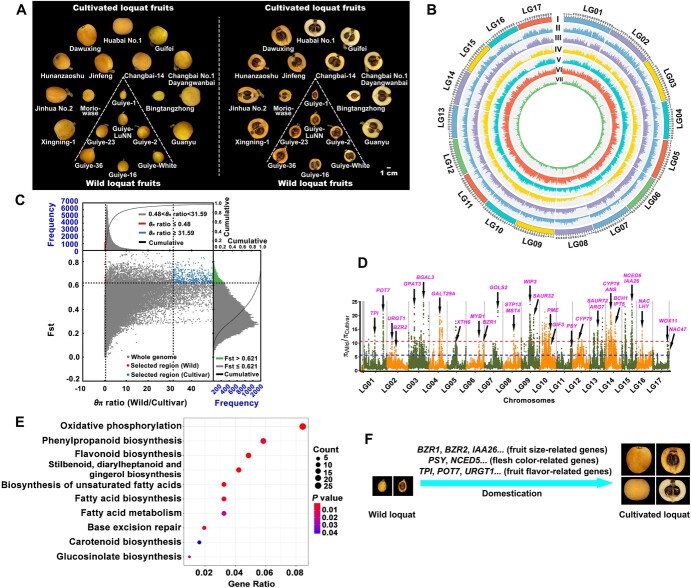
Whole-genome screen of selective sweep regions between wild and cultivated loquats. (A) Fruits of wild and cultivated loquats. Bar = 1 cm. (B) Circos demonstration of genetic diversity. (I) 17 pseudomolecules of wild loquat; (II) SNP density (numbers of SNPs in 1-Mb non-overlapping windows) of cultivated loquat Huabai No. 1; (III) SNP density of cultivated loquat ‘Jinhua No. 2’; (IV) SNP density of wild loquat GZ-23; (V) SNP density of wild loquat GZ-White; (VI) population differentiation (*F*_ST_) levels of wild loquat versus cultivated loquat; (VII) nucleotide diversity (π) of wild loquat versus cultivated loquat. (C) Whole-genome screen of selected regions according to the distribution of *θ*_π_ ratio and population differentiation (*F*_ST_). (D) Manhattan plots showing selective sweep regions in the loquat genome, based on the π ratio with 100-kb windows sliding in 10-kb steps. (E) KEGG pathway enrichment analysis of selected regions in the loquat genome, based on *F*_ST_. (F) Schematic of domestication in loquat fruit quality and flesh color.

Fruit quality and flesh color are important agricultural characteristics in loquat (Fig. 4A). The distributions and change trends of SNP density between wild and cultivated loquats were consistent, and the areas of high π values in the loquat genome were consistent with highly repetitive regions (Fig. 4B). The candidate selective sweeps were identified during the domestication process throughout the genome for all of the accessions in the germplasms, using nucleotide diversity and composite likelihood rates. Whole-genome scans of the wild loquat genome showed very few selected regions (Fig. 4C). However, a total of 283 selective sweep regions, covering 7.3% (57.46 Mb) of the assembled genome, and 2381 genes were identified in the selected regions of cultivated loquats (Supplementary Data Table S11). Among these selective sweep regions, functions of the selected genes mainly involved biosynthesis and metabolism of sugars, organic acids, fatty acids, amino acids, flavonoids, carotenoids, and plant hormones (Fig. 4D), which were associated with fruit quality and flesh color. Meanwhile, fruit size-related genes, such as *BZR1*, *BZR2*, *IAA26*, *NAC*, *SAUR32*, and *SAUR72*, were also under selection (Fig. 4D). Furthermore, enrichment analysis of the selective sweep genes were also mainly focused on the pathways of starch and sucrose metabolism, carbon metabolism, fructose and mannose metabolism, plant–pathogen interaction, fatty acid metabolism, phenylpropanoid biosynthesis, flavonoid biosynthesis, carotenoid biosynthesis, and plant hormone signal transduction (Fig. 4E and Supplementary Data Fig. S8). Taking these results together, we propose a unique domestication model for fruit quality and flesh color in loquat (Fig. 4F).

### Characterization of gene expression in loquat fruits

**Figure 5:**
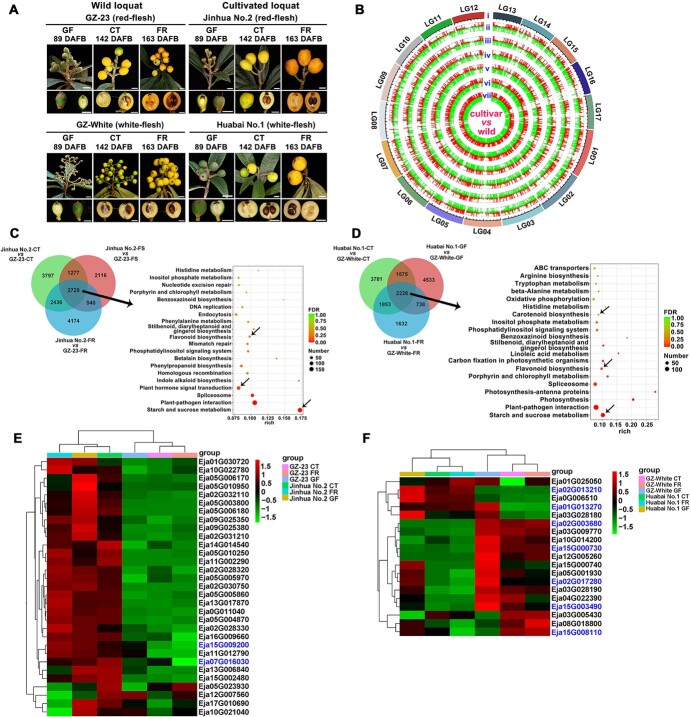
DEGs in loquat fruits. (A) Three fruit development stages in wild and cultivated loquats. GF, green fruit; CT, color turning; FR, fruit ripening. (B) Expression level changes of DEGs during the fruit development process in wild and cultivated loquats. (i) 17 pseudomolecules; (ii) DEGs in Huabai No. 1 GF versus GZ-White GF; (iii) DEGs in Huabai No. 1 CT versus GZ-White CT; (iv) DEGs in Huabai No. 1 FR versus GZ-White FR; (v) DEGs in Jinhua No. 2 GF versus GZ-23 GF; (vi) DEGs in Jinhua No. 2 CT versus GZ-23 CT; (vii) DEGs in Jinhua No. 2 FR versus GZ-23 FR. (C) Enrichment analysis of overlapping DEGs in Huabai No. 1 versus GZ-White. (D) Enrichment analysis of overlapping DEGs in Jinhua No. 2 versus GZ-23. (E) Heat map of starch and sucrose metabolism-related DEGs in Jinhua No. 2 versus GZ-23. (F) Heat map of flavonoid biosynthesis-related DEGs in Huabai No. 1 versus GZ-White. Upregulated and downregulated genes are indicated in red and green, respectively. IDs of key DEGs are indicated in blue.

Loquats are generally divided into white-fleshed and red-fleshed varieties. The fruits of cultivated loquats taste better than those of wild loquats, in both the white-fleshed and yellow-fleshed varieties (Fig. 4A). To determine the key regulatory genes involved in fruit quality, we performed comparative transcriptome analysis during three stages [i.e. green fruit (GF), color turning (CT), and fruit ripening (FR)] of fruit development and ripening. A total of 23 275 genes were expressed at the three developmental stages and 5435 genes were significantly changed, of which 1577 encoded transcription factors (TFs) from 56 families (Fig. 5A and B and Supplementary Data Tables S12 and S13). Among them, the 10 TF families with the highest number of genes, including MYB (170 members), ERF (114 members), bHLH (106 members), NAC (94 members), C2H2 (89 members), MIKC_MADS (64 members), WRKY (62 members), LBD (59 members), G2-like (55 members), and HD-ZIP (54 members), were identified. These differentially expressed genes (DEGs) may contribute to the unique fruit features and were mainly enriched in the pathways of starch and sucrose metabolism, plant hormone signal transduction, MAPK signaling pathway, glycolysis/gluconeogenesis, fatty acid elongation, circadian rhythm, flavonoid biosynthesis, fructose and mannose metabolism, carbon fixation in photosynthetic organisms, α-linolenic acid metabolism, photosynthesis, pentose phosphate pathway, porphyrin and chlorophyll metabolism, ubiquinone and other terpenoid-quinone biosynthesis, carotenoid biosynthesis, and others (Supplementary Data Fig. S9).

Compared with wild loquats, the DEGs in cultivated loquats were mainly classified in the pathways of carbohydrate metabolism, plant hormone signal transduction, flavonoid biosynthesis, and carotenoid biosynthesis, during fruit development (Fig. 5C and D). Among these DEGs, starch and sucrose metabolism-related genes Eja03G019190 *SUCS*, Eja07G016030 *TPS*, Eja09G025520 *SPS2*, and Eja15G009200 *SPS1* were significantly upregulated at the CT and FR stages in cultivated loquats (Fig. 5E and Supplementary Data Fig. S10). Plant hormone signal transduction-related genes Eja11G023360 *CRK2*, Eja12G011050 *EIN3*, Eja14G002840 *PYL1*, and Eja17G007830 *PYL4* were significantly upregulated at the CT stage in cultivated loquats, while Eja08G020920 *IAA32*, Eja02G013840 *AUX22*, Eja09G013660 *SAUR50*, Eja09G017740 *EIN2*, Eja17G021130 *ABI5*, Eja06G014810 *GAI*, and Eja16G014230 *ARF9* were significantly upregulated in wild loquats. Flavonoid biosynthesis-related genes Eja01G013270 *SALAT* and Eja02G013210 *BAHD1* were significantly upregulated in cultivated loquats, while Eja02G003680 *ANR*, Eja15G000730 *CYP98A*, Eja02G017280 *CYP73A*, Eja15G003490 *UGT88F*, and Eja15G008110 *FLS* were significantly upregulated in wild loquats (Fig. 5F and Supplementary Data Fig. S11). Meanwhile, we found that most carotenoid biosynthesis-related genes, including Eja03G021250 *DXR*, Eja03G015630 *GGPPS*, Eja13G012700 *PSY2B*, Eja04G021860 *PDS*, Eja16G003270 *BCH2*, Eja17G001130 *VDE*, and Eja06G006650 *CYP97C*, were significantly upregulated in red-flesh fruits compared with white-flesh fruits (Supplementary Data Fig. S12); this is consistent with the long-term understanding that the red flesh of loquat fruits is a result of carotenoid accumulation [20].

### Changes in metabolites in loquat fruits

**Figure 6:**
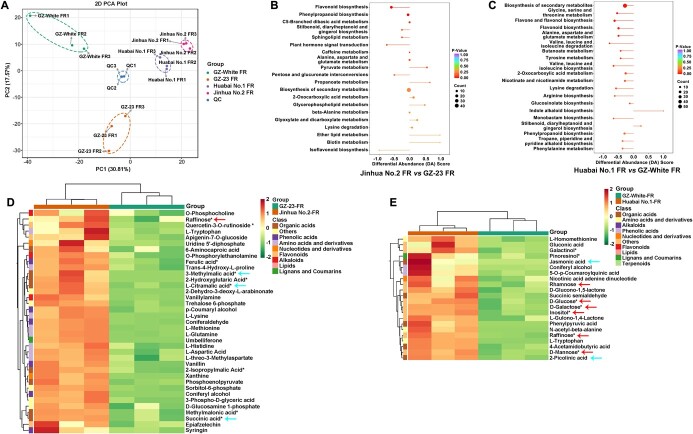
Metabolomic analysis of fruit ripening between wild and cultivated loquats. (A) PCA analysis of metabolites. (B) Enrichment analysis of DAMs in Jinhua No. 2 FR versus GZ-23 FR. (C) Enrichment analysis of DAMs in Huabai No. 1 FR versus GZ-White FR. (D) Heat map of DAMs in Jinhua No. 2 FR versus GZ-23 FR. (E) Heat map of DAMs in Huabai No. 1 FR versus GZ-White FR.

To determine metabolic changes at the FR stage, we performed widely targeted LC–MS/MS based on metabolite profiling in wild and cultivated loquats. A total of 1040 metabolites were identified, including 212 flavonoids, 197 phenolic acids, 127 lipids, 92 amino acids and derivatives, 77 organic acids, 69 saccharides and alcohols, 63 terpenoids, 52 nucleotides and derivatives, 51 alkaloids, 19 vitamins, and other metabolites (Supplementary Data Table S14). Hierarchical cluster analysis (HCA) and PCA of the metabolite profiles in four loquat fruits were classified them into four distinct groups (Fig. 6A and Supplementary Data Fig. S13), reflecting significant differences in metabolite profiles during the fruit development process between wild and cultivated loquats.

Metabolomic analysis of Jinhua No. 2 FR versus GZ-23 FR showed a total of 371 differentially accumulated metabolites (DAMs), of which 149 were upregulated, but 222 were downregulated. The regulatory pathways of DAMs were mainly involved in flavonoid biosynthesis; phenylpropanoid biosynthesis; C5-branched dibasic acid metabolism; stilbenoid, diarylheptanoid, and gingerol biosynthesis; plant hormone signal transduction; caffeine metabolism; alanine, aspartate, and glutamate metabolism; pyruvate metabolism; pentose and glucuronate interconversions; and others (Fig. 6B). Among these DAMs, raffinose was significantly upregulated in cultivated loquats. Among organic acids, 3-methylmalic acid, l-citramalic acid, and succinic acid were significantly upregulated in red-flesh cultivated loquats (Fig. 6D). However, some important flavonoids, including cynaroside, isoquercitrin, kaempferin, and astragalin, were significantly upregulated in wild loquats.

A total of 413 DAMs showed a significant difference upon comparison of Huabai No. 1 FR and GZ-White FR, of which 63 were upregulated and 350 were downregulated. The regulatory pathways of DAMs were mainly enriched in the biosynthesis of secondary metabolites; glycine, serine, and threonine metabolism; flavone and flavonol biosynthesis; flavonoid biosynthesis; alanine, aspartate, and glutamate metabolism; valine, leucine, and isoleucine biosynthesis; butanoate metabolism; and others (Fig. 6C). Among these DAMs, rhamnose, raffinose, d-glucose, d-galactose, d-mannose, and inositol were significantly upregulated in white-flesh cultivated loquats. Among organic acids, jasmonic acid and 2-picolinic acid were significantly upregulated in white-flesh cultivated loquats (Fig. 6E).

**Figure 7:**
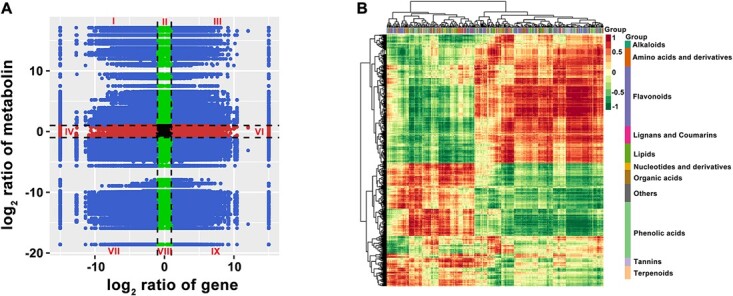
Correlation analysis of transcriptome and metabolome in ripening fruits. (A) Correlation analysis of transcriptome and metabolome in Jinhua No. 2 FR versus GZ-23 FR. I and IX, opposite change trend in DEGs and DAMs; III and VII, the same change trend in DEGs and DAMs; II, IV, VI and VIII, non-correlation in DEGs and DAMs. (B) Correlation coefficient clustering of the associated DEGs and DAMs in Jinhua No. 2 FR versus GZ-23 FR.

To further obtain an accurate understanding of the relationships between transcript levels and metabolite changes in ripening fruits, we carried out correlation analysis between DEGs and DAMs (Fig. 7A). Among them, the DEGs and DAMs with the same change trend were mainly involved in flavonoids, phenolic acids, amino acids and derivatives, terpenoids, organic acids, lipids, and others (Fig. 7B and Supplementary Data Fig. S14).

## Discussion

In the present study, we generated the first high-quality chromosome-level genome of wild loquat, which is 23–50 Mb larger than that of the sequenced genome of cultivated loquats [21–23], allowing us to investigate loquat evolution, domestication. and molecular breeding. The recent WGD in loquat must have occurred at 30–45 MYA, after the advent of the family Rosaceae (99–112.7 MYA) [19] but prior to the divergence from apple and pear (~17.6 MYA based on our results). The WGD events occurred throughout the routes of plant evolution and led to chromosome number evolution, gene expansion, and emergence of novel traits and functional divergence [24–26]. Previous studies have shown that a relatively recent WGD has led to the transition from 9 ancestral chromosomes to 17 chromosomes in the Pyreae, including *Eriobotrya*, *Malus*, and *Pyrus* [27]. Furthermore, the genetic consequences of WGD have increased adaptive plasticity and are responsible for the successful domestication of many plant crops [28]. In our study, we found that expanded gene families after the recent WGD in loquat were mainly involved in pollen recognition, tissue development, and the immune system, suggesting that the loquat WGD events induce gene expansion contributing to developmental processes and environmental adaptation. Previously, the extended gene families involved in photosynthesis and sugar metabolism have been found to be preferentially retained in mango [29]. In *Sesamum indicum*, the expansion genes are associated with oil biosynthesis and further facilitate the domestication with the aim of increasing oil content [30]. In Tibetan wheat, the expansion of α-gliadin genes represents genetic preconditions for the unique viscoelastic properties [31]. Taken together, these results indicate that WGD events have not only led to higher genetic diversity, but could also enhance adaptability to new environments in many crops.

The LD decay of the wild loquats was faster than that of cultivated loquats. The heterozygosity in cultivated germplasms was also significantly lower than that in wild germplasms, suggesting that the domestication process led to a decrease of genomic diversity in the cultivated loquats. Therefore, our results suggest that, during loquat breeding programs, wild germplasms provide a valuable genetic resource for the improvement of cultivated loquat. Furthermore, the LD decay in loquats is similar to that in other fruit trees and woody perennials, such as grapevine [9], pear [8], and apple [32], mainly due to their high heterozygosity and long generation time, but is much faster than that in annual crops such as rice [33], lupin [34], and soybean [13]. Compared with wild germplasms in crop species, cultivated germplasms have lower levels of genetic diversity, because of the strong impact of human selection [12, 13]. For example, genetic diversity in wild germplasms of tea tree is higher than that in cultivated germplasms, due to the reduction in genetic diversity during breeding practices and domestication [35]. Trait improvement has led to limited genetic diversity in commonly used breeding materials during apple domestication, suggesting a major effect of artificial selection in decreasing genetic diversity [32]. In this work, we further identified that the genes related to flavor, nutritional components, and fruit size have been under selection during the loquat domestication process. Similarly, lignin-, sugar-, and acid metabolism-related genes have been under continuous selection during pear improvement [8]. During peach domestication, fruit size and taste have been the predominant targets of selection and the related candidate genes have undergone selection [36].

Generally, the flavor of loquat fruit mostly depends on the balance between the compositions and contents of sugars and organic acids [37]. In accordance with the selective sweep regions, transcript levels of fruit quality, size, and flesh color-related genes showed significant differences between wild and cultivated loquats. Compared with wild loquats, some important sugar metabolism-related genes were significantly upregulated in cultivated loquats during fruit development. Similarly, a number of genomic regions in specific populations have been selected during crop domestication and this led to gene regulatory changes and phenotypic differences between different lineages [38–42]. For example, domestication selection causes loss of function of *TtBtr1* genes in domesticated emmer wheat and durum wheat [41]. However, the transcript abundances of expression quantitative trait loci are affected by positive selection in cultivated rice [42]. Furthermore, in our work, consistent with the changes in sucrose metabolism-related genes, saccharides were significantly accumulated in cultivated loquats, and the quantities of saccharides in the white-fleshed loquats were higher than those in yellow-fleshed loquats. Previous studies in cultivated loquats have indicated that the white-fleshed fruits have higher abundance of sugars and taste better than the yellow-fleshed fruits [4, 43]. Similarly, glucose is significantly accumulated along with starch degradation during the post-ripening stages in kiwifruits [44, 45]. Meanwhile, organic acids are related to fruit acidity and affect the taste of fruits [37, 46]. In our study, the contents of organic acids, such as citramalic acid and succinic acid, accumulated in cultivated loquats, suggesting that cultivated loquats have stronger taste. Previous studies have indicated that the components and content of organic acids contribute to the taste of longan fruits [47]. Besides sugars, organic acids have an impact on the good taste of strawberry fruits [46]. Furthermore, flavonoids are the key chemicals that contribute to the medicinal and nutritional characteristics of fruits [47]. In our work, most of the flavonoid biosynthesis-related genes were significantly upregulated in wild loquats (Supplementary Data Table S14). Accordingly, some high-value flavonoids, such as isoquercitrin and astragalin, were significantly accumulated in wild loquats, indicating that the fruits of wild loquats have higher medical value.

### Conclusions

We present what is, to our knowledge, the first high-quality chromosome-level genome of wild loquat. The complete genome sequence and whole-genome resequencing of loquats provide important resources that could aid in the exploration of loquat evolution and genetic diversity at the genome level. The identified SNP molecular markers should be used in advanced breeding programs and benefit the genome-enabled breeding of loquat. These genome sequences and multi-omics data will also serve as valuable resources for elucidating fruit domestication and the biosynthesis of secondary metabolites in loquat.

## Materials and methods

### Genome sequencing and assembly

Juvenile leaves were collected from wild loquat [Guiye-23 (GZ-23)] at the loquat germplasm resource nursery of Chongqing (29°80′ N, 106°40′ E). Total genomic DNA of the wild loquat GZ-23 was extracted from young fresh leaves, as described previously [48]. According to the protocols from Illumina (Illumina, San Diego, CA, USA), genomic DNA was then used to generate Illumina DNA libraries. For the genome survey, these short-read libraries were sequenced using the Illumina NovaSeq platform. Using the paired-end mode and ~ 150 bp read length, a total of 45.70 Gb Illumina short reads were generated, at ~62-fold genomic coverage. Based on *k*-mer distribution analysis (*k* = 19) using Jellyfish v2.1.3 [49], the genome size and heterozygosity ratio of wild loquat GZ-23 were estimated.

We extracted high molecular weight DNA and constructed Nanopore libraries according to the instructions of the Qiagen DNA purification kit (Qiagen, Germany) and the Ligation Sequencing Kit (SQK-LSK109). The Nanopore libraries were sequenced to generate Nanopore long reads using a PromethION platform (Oxford Nanopore Technologies Ltd, Oxford, UK), with the corresponding R9 cell and ONT sequencing reagents kit (EXP-FLP001.PRO.6). The Nanopore long reads were self-corrected using Canu [50]. Then, we assembled the corrected reads into contigs using SMARTdenovo (https://github.com/ruanjue/smartdenovo). Following this, initial assembled contigs were corrected using Racon and further polished using Medaka. Finally, the contigs were corrected again using Pilon [51], in conjunction with the Illumina short reads.

Hi-C fragment libraries of 300–700 bp were constructed using cross-linked DNA and sequenced using the platform of Illumina NovaSeq 6000. To obtain high-quality Hi-C data, adapter sequences were removed from the sequenced Hi-C reads and low-quality reads were filtered using Trimmomatic v0.36 [52]. Then, the cleaned Hi-C reads were mapped to the genome assembly using BWA [53]. Invalid read pairs were filtered using HiC-Pro v2.10.0 [54]. Meanwhile, the valid Hi-C read pairs (69.39% of uniquely mapped read pairs) were used to generate chromosome-scale scaffolds using LACHESIS [55]. After assembly, the completeness and accuracy of the genome was assessed using CEGMA [15] and BUSCO [56]. The quality of the wild loquat genome assembly was further assessed by mapping the RNA-seq reads from six tissues (root, stem, leaf, flower, fruit, and seed) back to the scaffolds, which supported the fact that the wild loquat genome assembly was of high quality.

### Repeat sequences and genome annotation of wild loquat

The repetitive sequences of loquat were identified before gene annotation using a combination of homology-based and *de novo* approaches. First, a *de novo* repeat library was built using RepeatModeler [57], which includes RECON [58] and RepeatScout [59]. Second, the repeat sequences were classified using RepeatClassifier [57], which includes RepBase [60], REXdb [61], and Dfam [62]. Third, the LTR retrotransposons were identified using LTR_retriever, with LTRharvest [63] and LTR_FINDER [64]. To construct the final non-redundant repetitive sequence database, the redundancies were filtered out and then integrated together with the repeats in RepBase [60]. Finally, the repetitive sequences were predicted by means of a homology search against the constructed library using RepeatMasker [65].

Protein-coding genes of the loquat genome were annotated using three different strategies: *ab initio* prediction, homology prediction, and transcriptome analysis methods. Augustus [66] and SNAP [67] were used for *ab initio* prediction with the self-trained parameters. Homology-based prediction was carried out by aligning peptide sequences from four species (*A. thaliana*, *M. domestica*, *P. bretschneideri*, and *F. vesca*) to the loquat genome assembly, using GeMoMa [68]. For transcript-based prediction, the clean RNA-seq reads of the whole plant were mapped to the loquat genome assembly using HISAT [69], and assembled into transcripts using StringTie [69, 70]. Based on the mapping transcripts, the RNA-seq reads were assembled and used to predict genes using PASA [71]. Finally, the obtained prediction results of the *ab initio* prediction, homology prediction, and transcriptome analysis, were integrated using EVM [71].

Functional annotations were performed by blasting the sequences of the predicted genes against biological databases, including NCBI-nr, Swiss-Prot, KEGG, KOG, GO, Pfam, and TrEMBL, with an *E*-value cutoff of 1e^−5^. The whole genomes were scanned to identify possible homologous gene sequences using GenBlastA, following which the frameshift mutations and premature termination codons in the gene sequences were identified as pseudogenes using GeneWise [72]. In addition, non-coding RNAs, including rRNA, miRNA, tRNA, snRNA, and snoRNA, were predicted. Among them, miRNA and tRNA were predicted using miRBase (release 21) and tRNAscan-SE [73]. The snRNA and snoRNA genes were predicted by blasting the genome sequences against the Rfam database using Infernal [74].

### Comparative genome analysis

Orthologous gene families from loquat and 12 other representative plant species (*A. chinensis*, *A. thaliana*, *C. sinensis*, *D. catenatum*, *F. vesca*, *M. domestica*, *N. colorata*, *O. sativa*, *P. bretschneideri*, *V. vinifera*, *S. lycopersicum*, and *M. indica*) were identified using OrthoFinder [75]. Then, we used the PANTHER database [76] to annotate the obtained orthologous gene families. Following this, KEGG and GO enrichment analyses of gene families unique to loquat were carried out using ClusterProfile [77].

We constructed a phylogenetic tree using IQ-TREE v1.6.11 [78] using the maximum likelihood method and 1000 bootstrap replicates. Divergence times of plant species were calculated using PAML v4.9i [79] with the MCMCTree package. The iteration number sets of the Markov chain were a burn-in number of 5 000 000, a sampfreq of 30, and an nSample of 5 000 000. Based on the phylogenetic tree with divergence time, we analyzed the number of gene family members in each branch using a birth mortality model. From the phylogenetic tree, the expansion and contraction of the gene families in loquat were examined relative to those in their ancestors using CAFE v4.2 [80]. Significant expansion or contraction was identified based on a *P*-value cutoff of .05. The identified expansion and contraction of gene families were annotated using PANTHER [76], following which the KEGG and GO enrichment analyses of these expansion and contraction gene families were identified using ClusterProfile [77].

### Analysis of WGD events

Means of all-against-all homology searches were used to determine homologous gene pairs by BLASTP (*E*-value threshold of 1e^−5^ and *C* score >.5). To detect homologous gene pairs, we used MCScanX [81] to detect syntenic blocks within a genome or between different genomes. Then, in these syntenic blocks, 4-fold synonymous third-codon transversion rates (4DTv) of orthologous/paralogous genes were calculated. Following this, the WGD events in loquat were estimated using 4DTv. We further used PAML [79] to calculate synonymous substitution (*K*_s_) values of paralogous blocks in wild loquat, and then the WGD events were detected by estimating the distribution of *K*_s_ values using wgd v1.1.1 [82], with default settings.

### Population genetics analysis

The high-quality resequencing reads from wild and cultivated loquats (~22.49× genomic coverage for each genome) were obtained using the Illumina HiSeq X™ Ten platform. All clean reads were mapped to the wild loquat genome using the MEM algorithm of BWA [53]. Genome alignment from each accession was carried out by marking duplicated reads using SAMtools [83] and Picard tools v1.94 (http://broadinstitute.github.io/picard/). Realignment in InDel regions was performed using InDel-Realigner in GATK [84]. The SNPs were further filtered and the SNPs with minor allele frequency ≥.05 and missing rates <.3 were considered high-quality SNPs. After filtration, SNP annotation was carried out using SnpEff software [85].

A phylogenetic tree of 37 loquat accessions was constructed and visualized using MEGA v6.0 [86] with the neighbor-joining method and 1000 bootstrap replicates. Population structure was performed using ADMIXTURE [87]. PCA was carried out to evaluate the genetic structure using smartPCA in EIGENSOFT [88]. LD was calculated using PLINK software (www.cog-genomics.org/plink2). The LD coefficient (*r*^2^) was analyzed for all chromosomes with a 1000-kb window. Based on genome-wide high-confidence SNPs, a sliding window approach with a 10-kb window step size and a 100-kb window size was used to calculate the values of the population differentiation statistic (*F*_ST_), nucleotide diversity ratio (*π*), and polymorphism level (*θ*_π_) using VCFtools [89].

### Transcriptome analysis

Wild and cultivated loquat fruits at three development stages, including GF, CT, and FR, were collected for transcriptome analysis. Total RNAs of loquat fruits of three independent biological replicates were extracted individually from each development stage using the RNAprep Pure Plant Plus Kit (DP441, Tiangen Biotech, China). Then, the concentration and integrity of total RNA were detected using NanoDrop™ 2000 (Thermo Scientific, USA) and an Agilent Bioanalyzer 2100 System (Agilent Technologies, USA). According to the protocols of the RNase-free DNase I (Takara) and TruSeq™ RNA Sample Preparation Kit (Illumina), the purified RNA was used to construct cDNA libraries. After end-pairing and ligation of sequencing adapters, the fragments were amplified and then sequenced using an Illumina HiSeq X™ Ten platform, which was provided by Shanghai Personal Biotechnology Co., Ltd (China). In total, 240 Gb raw data of RNA-seq were generated from 36 libraries.

Low-quality reads were filtered from the RNA-seq raw data, resulting in 221.2 Gb of clean reads. Then, we aligned the clean reads to the genome assembly of wild loquat using HISAT2 [70]. Fragments per kilobase of transcript per million mapped fragments (FPKM) values were calculated for DEGs in different tissues at different developmental stages. The DESeq program was used to analyze the DEGs [90]. Genes showing a |log2 ratio| >1 and adjusted *P* values <.01 were considered to be significant DEGs.

### Metabolite analysis

Metabolites of fruit samples were extracted according to the method described previously [91]. Metabolites extracted from loquat fruits were absorbed on a CNWBOND Carbon-GCB SPE Cartridge (250 mg, 3 ml; Anpel, Shanghai, China) and filtered using a 0.22-μm nylon syringe filter (SCAA-104, Anpel). Quality control of loquat fruit samples was performed by pooling an aliquot of wild and cultivated loquat fruit samples; this enabled the reproducibility of the mass spectrometric results. Following this, the phytochemical profiles of the loquat fruit extracts were determined using an UPLC-ESI-MS/MS system (UPLC, Shim-pack UFLC Shimadzu CBM30A system, Shimadzu, Japan). MS data were acquired using an Applied Biosystems 4500 Q Trap (AB Sciex, USA) and processed as described previously [92]. Then, the metabolite compounds were identified using the Metware in-house MS^2^ spectral tag (MS2T) library (http://www.metware.cn), which was provided by Wuhan Metware Biotechnology (Wuhan, China).

PCA and HCA of metabolites were carried out using the R package. Metabolites between groups showing absolute log_2_ fold change ≥1 and variable importance in projection (VIP) ≥0.8 were considered to be significantly regulated. From orthogonal partial least-squares discriminant analysis (OPLS-DA), the values of VIP were extracted, and then generated using MetaboAnalystR. The log transform (log_2_) and mean of the data were centered before OPLS-DA. Meanwhile, we used a permutation test (200 permutations) to avoid overfitting. Then, the identified metabolites were further annotated and enriched using the KEGG database [93]. Based on metabolite set enrichment analysis (MSEA), the pathways of differentially accumulated metabolites were performed. Furthermore, the significance of enrichment terms was calculated using the hypergeometric test’s *P*-value.

## Acknowledgements

This work was supported by the National Key R&D Program of China (No. 2019YFD1000200), the National Nature Science Foundation of China (No. 32102321), the Chongqing Science and Technology Commission (cstc2021jcyj-msxmX1156 and cstc2021jscx-gksbX0010), the Innovation Research Group Funds for Chongqing Universities (CXQT19005), and the Fundamental Research Funds for the Central Universities (SWU-KT22055). We thank Chao Nie (College of Horticulture and Landscape Architecture, Southwest University) for support during metabolite analysis. We also thank the BMKCloud (http://www.biocloud.net), which provided bioinformatics support in this project.

## Author contributions

D.J., Q.H., Q.G., and G.L. conceived and designed the project. D.J., X.L. and Q.H. performed the data analysis. D.J., J.D., Y.X., D.W., C.Z., Q.G., and G.L. collected the samples of loquat germplasms. X.L., R.H., and Y.Y. collected the samples of fruits. S.W. and Y.Z. contributed to valuable discussions. D.J. and Q.X. analyzed the karyotype of wild loquat. D.J. wrote the manuscript. X.L., Q.G., and G.L. revised the manuscript. All authors read and approved the final manuscript.

## Data availability

The raw genome sequence data have been deposited in the National Genomics Data Center (NGDC, https://ngdc.cncb.ac.cn/) under BioProject accession number PRJCA008992. The whole genome sequence of wild loquat has been deposited in the Genome Warehouse (https://ngdc.cncb.ac.cn/gwh/) under accession number GWHBOTF00000000.

## Conflict of interest

The authors declare that they have no conflict of interest.

## Supplementary data

Supplementary data is available at *Horticulture Research* online.

## Supplementary Material

Web_Material_uhac265
