# Supplementary material for: Genome assembly of wild loquat (Eriobotrya japonica) and resequencing provide new insights into the genomic evolution and fruit domestication in loquat
Source: Hortic Res. 2022 Dec 2;10(2):uhac265. doi: 10.1093/hr/uhac265 (PMC9909508; doi:10.1093/hr/uhac265)
Supplement: Web_Material_uhac265 [file web_material_uhac265.zip › Supplemental Figures.doc]

Supplemental Information

Figure S1 The chromosome survey of wild loquat. (A) Cytological detection of wild loquat chromosomes, which contain seventeen distinct pairs of chromosomes (2n=34). (B) Fow cytometry analysis of loquats, which was consistent with the size of ~760 Mb.

Figure S2 Biological process classification of specific gene families in loquat

Figure S3 Distribution of four-fold degenerate sites (4DTv) of duplicate gene pairs in wild loquat

Figure S4 Distribution of synonymous substitutions (Ks) of duplicate gene pairs in wild loquat and pear. (A) Ks distributions of duplicate gene pairs in wild loquat (*E.japonica*_*E.japonica*). (B) Ks distributions of duplicate gene pairs in pear (*P. bretschneideri*_ *P. bretschneideri*).

Figure S5 Phylogenetic tree of wild loquat and 12 other species based on a concatenated alignment of 1028 single-copy orthologs. The tree is rooted with *N. colorata* as the outgroup. The green and pink numbers indicate expanded and contracted gene families, respectively.

Figure S6 KEGG pathway enrichment analysis of the expanded gene families

Figure S7 Gene Ontology (GO) enrichment analysis of the contracted gene family

Figure S8 Enrichment analysis of top 1% of selective regions in cultivar *vs* wild.

Figure S9 KEGG pathway enrichment analysis of differentially expressed genes (DEGs) during fruit development.

Figure S10 Heat map of starch and sucrose metabolism-related DEGs in Huabai No.1 *vs* GZ-White. The up-regulated and down-regulated genes are indicated in red and green, respectively. The gene IDs of key DEGs are indicated in blue.

Figure S11 Heat map of plant hormone signal transduction-related DEGs in Jinhua No.2 *vs* GZ-23. The up-regulated and down-regulated genes are indicated in red and green, respectively. The gene IDs of key DEGs are indicated in blue.

Figure S12 Heat map of carotenoid biosynthesis-related DEGs in loquats. The up-regulated and down-regulated genes are indicated in red and green, respectively.

Figure S13 Hierarchical cluster analysis of the identified metabolites in fruit ripening in wild and cultivated loquats. The up-regulated and down-regulated metabolites are indicated in red and green, respectively.

Figure S14 Correlation analysis and correlation coefficient clustering of transcriptome and metabolome in Huabai No.1-FR and GZ-White-FR. (A) Correlation analysis of transcriptome and metabolome in Huabai No.1-FR *vs.* GZ-White-FR. I and IX: opposite change trend in DEGs and DAMs; III and VII: the same change trend in DEGs and DAMs; II, IV, VI and VIII: non-correlation in DEGs *vs.* DAMs. (B) Correlation coefficient clustering of the associated DEGs and DAMs in Huabai No.1-FR *vs.* GZ-White-FR.
