# Supplementary material for: Genome assembly of wild loquat (Eriobotrya japonica) and resequencing provide new insights into the genomic evolution and fruit domestication in loquat
Source: Hortic Res. 2022 Dec 2;10(2):uhac265. doi: 10.1093/hr/uhac265 (PMC9909508; doi:10.1093/hr/uhac265)
Supplement: Web_Material_uhac265 [file web_material_uhac265.zip › Supplemental Table S1-7.doc]

Supplemental Tables

Table S1 Genome survey sequencing of wild loquat

| Species | Fragment size of DNA libraries | Sequencing data | Depth | Estimated size of genome |
| --- | --- | --- | --- | --- |
| Wild loquat | 350 bp | 45.70 Gb | 62× | 737.06 Gb |

Table S2 Summary of genome sequencing data for wild loquat

| Seqencing Platform | Reads Number of Raw Data | Total Length of Raw Data |
| --- | --- | --- |
| Nanopore | 6,880,906 | 159.8 Gb |

Table S3 Summary statistics of sequencing data and an initial assembly of the Nanopore reads

| Data  Type | Sequencing Number | Total Length | N50 Length | N90 Length | Mean Length | Max Length | Depth |
| --- | --- | --- | --- | --- | --- | --- | --- |
| Clean data of Nanopore | 5,976,012 | 152.6 Gb | 31,640 | 15,117 | 25,538 | 260,195 | ~198.5× |
| Data  Type | Sequencing Number | Total Length | GC(%) | N(%) | Q20(%) | Q30(%) |  |
| Clean data of Hi-C | 270,608,968 | 81.06 Gb | 42.76 | 0.00 | 97.18 | 92.38 |  |
| Type | Contig length (bp) | Contig N50 (bp) | Contig N90 (bp) | Contig max (bp) | GC content (%) | Gap total length (bp) |  |
| Initial assembly | 768,750,576 | 4,391,035 | 889,434 | 16,605,339 | 37.81 | 0 |  |

Table S4 Assessment of transcripts mapping to the assembly

| Data Type | Raw Data | Clean Data | Mapped (%) |
| --- | --- | --- | --- |
| RNA-Seq transcripts | 9.56 Gb | ~8 Gb | 98.7 |

Table S5. Summary of transposable elements in wild loquat genome

| Type | Number | Length | Rate (%) |
| --- | --- | --- | --- |
| ClassI: Retroelement | 485,609 | 399,515,718 | 50.92 |
| ClassI/ClassI/LTR/Cassandra | 15 | 1,220 | 0.00 |
| ClassI/ClassI/LTR/Caulimovirus | 1,927 | 2,506,187 | 0.32 |
| ClassI/ClassI/LTR/Copia | 121,656 | 97,782,487 | 12.46 |
| ClassI/ClassI/LTR/Gypsy | 148,907 | 188,562,341 | 24.03 |
| ClassI/ClassI/LTR/Pao | 2,206 | 563,395 | 0.07 |
| ClassI/ClassI/LTR/Unknown | 154,960 | 96,950,955 | 12.36 |
| ClassI/ClassI/LTR/Viper | 63 | 7,873 | 0.00 |
| ClassI/DIRS | 597 | 48,261 | 0.01 |
| ClassI/LINE | 37,296 | 9,776,591 | 1.25 |
| ClassI/LTR/ERV | 9,172 | 628,284 | 0.08 |
| ClassI/LTR/Unknown | 4,314 | 1,944,381 | 0.25 |
| ClassI/SINE | 4,496 | 743,743 | 0.09 |
| ClassII: DNA transposon | 265,114 | 69,324,287 | 8.83 |
| ClassII/Academ | 58 | 7,068 | 0.00 |
| ClassII/CACTA | 22,091 | 7,349,217 | 0.94 |
| ClassII/Crypton | 869 | 55,343 | 0.01 |
| ClassII/Dada | 830 | 117,757 | 0.02 |
| ClassII/Ginger | 310 | 18,632 | 0.00 |
| ClassII/Helitron | 10,107 | 3,957,926 | 0.50 |
| ClassII/IS3EU | 222 | 11,501 | 0.00 |
| ClassII/Kolobok | 763 | 73,607 | 0.01 |
| ClassII/MITE | 35 | 2,126 | 0.00 |
| ClassII/Maverick | 648 | 48,835 | 0.01 |
| ClassII/Merlin | 91 | 4,556 | 0.00 |
| ClassII/Mutator | 41,449 | 8,896,296 | 1.13 |
| ClassII/Novosib | 470 | 48,751 | 0.01 |
| ClassII/P | 275 | 13,971 | 0.00 |
| ClassII/PIF-Harbinger | 30,514 | 10,661,255 | 1.36 |
| ClassII/PiggyBac | 502 | 29,865 | 0.00 |
| ClassII/Sola | 358 | 24,984 | 0.00 |
| ClassII/Tc1-Mariner | 3,586 | 252,607 | 0.03 |
| ClassII/Unknown | 102,790 | 21,242,273 | 2.71 |
| ClassII/Unknown | 1 | 59 | 0.00 |
| ClassII/Zator | 28 | 1,731 | 0.00 |
| ClassII/Zisupton | 496 | 32,236 | 0.00 |
| ClassII/hAT | 48,621 | 16,473,691 | 2.10 |
| Total | 750,723 | 468,840,005 | 59.75 |

Table S6. Summary of non-coding RNA in wild loquat genome

| rRNA number | tRNA number | miRNA number | snRNA number | snoRNA number |
| --- | --- | --- | --- | --- |
| 5,381 | 765 | 129 | 143 | 954 |

Table S7. Clustering statistics of gene family in loquat and 12 other plant species

| Item | A.chi | A.tha | C.sin | D.cat | E.jap | F.ves | M.dom | M.ind | N.col | O.sat | P.bre | S.lyc | V.vin |
| --- | --- | --- | --- | --- | --- | --- | --- | --- | --- | --- | --- | --- | --- |
| Number of genes | 33,043 | 27,589 | 36,951 | 29,149 | 45,791 | 28,588 | 45,116 | 34,522 | 20,066 | 38,714 | 34,569 | 34,359 | 26,346 |
| Number of genes in orthogroups | 31,002 | 18,623 | 25,314 | 23,035 | 42,150 | 22,384 | 37,683 | 27,923 | 18,107 | 21,847 | 33,150 | 23,506 | 20,112 |
| Number of unassigned genes | 2,041 | 8,966 | 11,637 | 6,114 | 3,641 | 6,204 | 7,433 | 6,599 | 1,959 | 16,867 | 1,419 | 10,853 | 6,234 |
| Percentage of genes in orthogroups | 93.8 | 67.5 | 68.5 | 79.0 | 92.0 | 78.3 | 83.5 | 80.9 | 90.2 | 56.4 | 95.9 | 68.4 | 76.3 |
| Percentage of unassigned genes | 6.2 | 32.5 | 31.5 | 21.0 | 8.0 | 21.7 | 16.5 | 19.1 | 9.8 | 43.6 | 4.1 | 31.6 | 23.7 |
| Number of orthogroups containing species | 17,036 | 12,801 | 16,543 | 13,632 | 20,668 | 16,630 | 20,527 | 16,523 | 12,722 | 13,034 | 16,874 | 15,874 | 15,354 |
| Percentage of orthogroups containing species | 38.7 | 29.1 | 37.6 | 31.0 | 47.0 | 37.8 | 46.6 | 37.5 | 28.9 | 29.6 | 38.3 | 36.1 | 34.9 |
| Number of species-specific orthogroups | 276 | 2,430 | 979 | 1,449 | 523 | 750 | 430 | 836 | 423 | 2,928 | 197 | 2,076 | 511 |
| Number of genes in species-specific orthogroups | 699 | 6,573 | 2,863 | 5,804 | 1,778 | 2,398 | 1,163 | 2,447 | 1,617 | 8,581 | 522 | 6,581 | 1,580 |
| Percentage of genes in species-specific orthogroups | 2.1 | 23.8 | 7.7 | 19.9 | 3.9 | 8.4 | 2.6 | 7.1 | 8.1 | 22.2 | 1.5 | 19.2 | 6.0 |

A. chi: *Actinidia chinensis*; A. tha: *Arabidopsis thaliana*; C. sin: *Camellia sinensis*; D. cat: *Dendrobium catenatum*; E. jap: *Eriobotrya japonica*; F. ves: *Fragaria vesca*;	M. dom: *Malus domestica*;	M. ind: *Mangifera indica*; N. col: *Nymphaea colorata*; O. sat: *Oryza sativa*; P. bre: *Pyrus bretschneideri*; S.lyc: *Solanum lycopersicum*; V. vin: *Vitis vinifera*
